# Evaluation of site-specific homologous recombination activity of BRCA1 by direct quantitation of gene editing efficiency

**DOI:** 10.1038/s41598-018-38311-x

**Published:** 2019-02-07

**Authors:** Yuki Yoshino, Shino Endo, Zhenghao Chen, Huicheng Qi, Gou Watanabe, Natsuko Chiba

**Affiliations:** 1 Department of Cancer Biology, Institute of Development, Aging and Cancer, Tohoku University, 4-1 Seiryomachi, Aoba-ku, Sendai, 980-8575 Japan; 2 Tohoku Medical and Pharmaceutical University, 1-12-1 Fukumuro, Miyagino-ku, Sendai, 983-8512 Japan

## Abstract

Homologous recombination (HR) contributes to the repair of DNA double-strand breaks (DSBs) and inter-strand crosslinks. The HR activity in cancer cells can be used to predict their sensitivity to DNA-damaging agents that cause these damages. To evaluate HR activity, we developed a system called Assay for Site-specific HR Activity (ASHRA), in which cells are transiently transfected with an expression vector for CRISPR/Cas9 and a HR donor sequence containing a marker gene. DSBs are created by Cas9 and then repaired by HR using donor vector sequences homologous to the target gene. The level of genomic integration of the marker gene is quantified by Western blotting, flowcytometry, or quantitative PCR (qPCR). ASHRA detected HR deficiency caused by BRCA1, BARD1, or RAD51 knockdown or introduction of BRCA1 variants. The influence of BRCA1 variants on HR, as determined by qPCR, was consistent with the chemosensitivities of the transfected cells. The qPCR format of ASHRA could measure HR activity in both transcribed and un-transcribed regions. Knockdown of BRCA1 nor BARD1 did not affect HR activity in a transcriptionally inactive site. ASHRA can evaluate HR activity and will be useful for predicting sensitivity to chemotherapy, screening drugs that affect HR, and investigating the mechanisms of HR.

## Introduction

The causes of DNA damage include chemicals, ionizing radiation, replication errors, and mitotic errors^1^. DNA double-strand breaks (DSBs) are the most deleterious kind of DNA damage. Accordingly, cells have two major pathways for repair of DSBs: homologous recombination (HR) and non-homologous end joining (NHEJ)^1,2^. HR operates in late S/G2 phase of the cell cycle, using the sister chromatid as a recombination template. By contrast, NHEJ, which repairs DSBs by direct joining, is error-prone and frequently causes deletion or insertion of DNA around the DSBs^3^. Consequently, HR is more important for maintaining genomic integrity and suppressing carcinogenesis^4–6^.

HR deficiency confers sensitivity to some types of cancer chemotherapy. For example, DNA-damaging agents such as camptothecin, etoposide, and ionizing radiation create DSBs^7–10^. Platinum compounds produce inter-strand crosslinks, repair of which also requires HR activity^3,11^. Accordingly, HR deficiency increases susceptibility to these DNA-damaging agents. Recently, poly (ADP-ribose) polymerase (PARP) inhibitors, which cause synthetic lethality in HR-deficient cells, have been developed and applied in the clinic^12–16^. Evaluation of the HR activity in cancer cells will be useful for stratifying cancer patients and identifying those who are likelier to respond to the treatment with DNA-damaging agents and PARP inhibitors.

HR deficiency is caused by derangements of various genes^17–19^. *BRCA1* and 2, which are the responsible genes for hereditary breast and ovarian cancer syndrome (HBOC), are the critical factors of HR^3,20^. In breast or ovarian cancers in HBOC patients, expression of wild-type BRCA1/2 is frequently eliminated due to loss of heterozygosity^20^. Such cancers are highly sensitive to platinum compounds^11,21–23^, ionizing radiation^10,24,25^, and PARP inhibitors^13–16^. However, secondary mutation^26^ or upregulation^27^ of BRCA1 can lead to secondary resistance to chemotherapy. Therefore, the mutation status of *BRCA1/2* is insufficient to stratify patients. In addition, not all patient-derived *BRCA1/2* variants result in HR deficiency^22,28,29^. Furthermore, HR is impaired by derangement of not only BRCA1/2, but also other HR factors. Indeed, as much as half of the HR deficiency in all cancers is due to derangement of factors other than BRCA1/2^19,30^. Therefore, evaluation of HR activity itself is important for the prediction of sensitivity to these agents.

Several approaches for estimating cellular HR activity have been developed. One example is the HR deficiency score (HRD score), which is calculated from the number of genetic alterations caused by HR deficiency. In ovarian cancers, the HRD score is correlated with sensitivity to cisplatin^31^. However, the HRD score does not evaluate HR activity itself, and is therefore inappropriate for studies of HR pathways or drug screening, in which changes of HR activity must be evaluated over short periods of time.

Another assay method, the direct-repeat GFP (DR-GFP) assay, uses genetically modified cell lines^29,32,33^ in which two incomplete GFP cassettes are stably integrated into the genome. In the first cassette, the GFP gene has a promoter, but contains a premature stop codon and the I-SceI restriction site, and is therefore non-functional. The second cassette has an intact coding sequence but lacks a promoter. In HR-proficient cells, a DSB created by I-SceI in the first cassette is repaired by HR using the second cassette as a template, yielding an intact GFP gene with a functional promoter. To estimate HR activity, GFP-positive cells are counted by flow cytometry (FC). The DR-GFP assay has been widely used to evaluate HR activity. However, this assay measures HR activity in a foreign gene, rather than in endogenous genes. Of greater concern, HR activity determined by the DR-GFP assay is sometimes poorly correlated with sensitivity to anti-cancer agents. Our group and others have analyzed various BRCA1 variants by DR-GFP assay^22,28,29,34–36^. In addition, some of these BRCA1 variants result in elevated sensitivity to DNA-damaging drugs, including cisplatin and PARP inhibitors^12–16,23,35–38^. These results revealed that BRCA1 variants like I26A exhibit only a mild decrease in HR activity, but confer high sensitivity to DNA-damaging drugs and PARP inhibitors^22^. Therefore, it is possible that HR activities determined by DR-GFP assay are inconsistent with sensitivity to DNA-damaging agents, at least in some cases.

As a modification of the DR-GFP assay, Pinder *et al*. used a reporter knock-in assay using CRISPR/Cas9 to estimate HR activity^39^. In their assay, a specific DSB is created in a target gene by a mutant Cas9 with nickase activity; this is done using two guide RNAs (gRNA) designed to bind in close proximity to the sense and anti-sense strands. The DSB is then repaired by HR using a HR donor vector that contains sequences homologous to the target gene and mClover as a reporter. Cells that express the target::mClover fusion gene are counted by FC. Although this assay enables evaluation of HR in endogenous gene loci, it cannot detect HR activity in genomic regions that are un-transcribed or expressed at very low levels.

Recently, Aymard *et al*. reported that epigenetic modifications of histones control the distribution of DNA repair factors along DNA strands^40^. In contrast to NHEJ-related factors, which are distributed evenly throughout the genome, HR-related factors localize mainly to transcriptionally active regions. Accordingly, it is reasonable to speculate that HR is more active in transcribed than un-transcribed regions. This suggests that HR activity might depend on the locus selected for measurement. To date, however, existing methods have been unable to evaluate HR activity in non-expressed loci.

In this study, we developed an assay for evaluating cellular HR activity using the CRISPR/Cas9 system. The new assay can estimate HR activity directly on the DNA level, with high sensitivity, in any type of sample, in any regions in the genome, and irrespective of transcriptional activity. The HR activities of BRCA1 variants determined by this assay were better correlated with sensitivity to anti-cancer agents than those estimated by the DR-GFP assay.

## Results

### Specific detection of HR products after transient transfection

**Figure 1:**
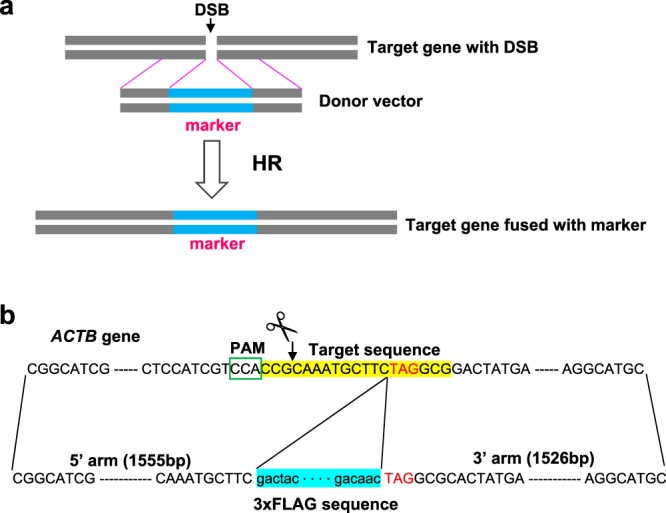
Scheme of knock-in of a marker sequence into an endogenous target gene by gene editing. (**a**) Illustration of the knock-in. (**b**) Structure of the donor vector containing 3xFLAG sequence between the homology arms for the *ACTB* gene. The target sequence of the gRNA, highlighted in yellow, is placed on the anti-sense strand around the stop codon (highlighted by red color) of *ACTB*. The DSB is created 10 bp upstream from the stop codon and 3 bp downstream of the PAM sequence (indicated by the scissors). The donor vector contains 5′ and 3′ arms and a marker sequence, highlighted in blue, just upstream of the stop codon.

In our assay, cells are transiently transfected with two vectors for expression of gRNA and Cas9 and HR donor sequence. After transfection, DSBs in the genome are created by Cas9 at the gRNA target site. DSBs are repaired by HR activity using the donor vector, which contains a marker sequence inserted between arm sequences homologous to the target site (Fig. 1a). The marker sequences in the donor vector are integrated into the genome, and HR activity is estimated based on the amount of the marker sequence in the genome.

We chose the β-actin gene (*ACTB*) as the target because its expression is high and stable in various cell types. The gRNA sequence spanned the stop codon of *ACTB*, thus directing Cas9 to cut near the stop codon (Fig. 1b). gRNA with a scrambled sequence was used as a control. The sequences of these gRNAs were cloned into LentiCRISPR v2 to yield ACTB-Cas9 and scr-Cas9, respectively; both constructs simultaneously express wild-type Cas9 and the gRNA. To construct HR donor vectors, the sequence from −1554 to +1527 bp from the stop codon of *ACTB* was cloned into pBluescript SK II+, and the sequences encoding 3xFLAG or mClover (as a marker) were inserted just 5′ of the stop codon of *ACTB* (Fig. 1b).

**Figure 2:**
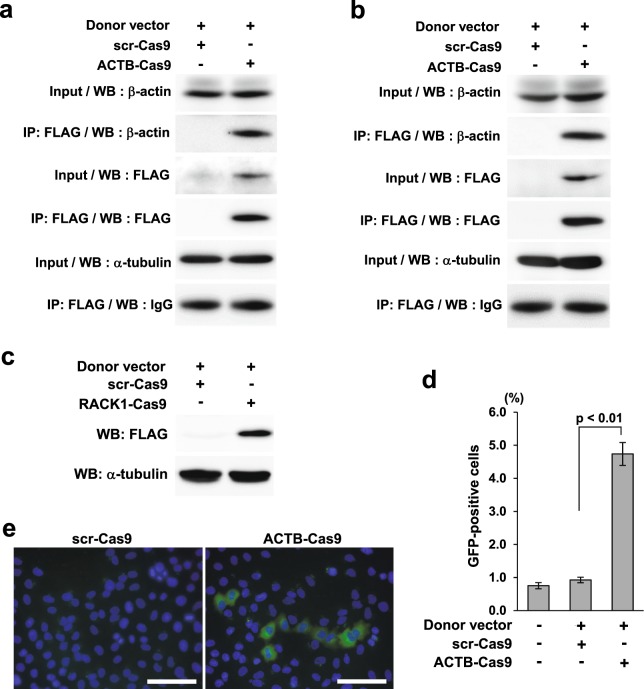
Detection of the fusion gene product of the marker and β-actin. (**a**) Detection of β-actin::3xFLAG fusion gene product in HeLa cells by IP.The donor vector (0.5 μg) and ACTB-Cas9 or scr-Cas9 (0.5 μg) were transfected into HeLa cells in 3.5 cm dishes. Cells were harvested 72 hours after transfection. Lysates and proteins immunoprecipitated with anti-FLAG antibody were analyzed by WB. α-tubulin or IgG was used as a loading control for the input or IP samples respectively. (**b**) Detection of β-actin::3xFLAG fusion gene product in HEK-293T cells by IP. Samples were prepared in the same way as (**a**). (**c**) WB of RACK1::3xFLAG fusion protein in HeLa cells. Cells were harvested for WB 72 hours after transfection of RACK1-Cas9 and donor vectors. (**d**,**e**) Detection of β-actin::mClover fusion gene by FC and fluorescent microscopy. Error bars indicate standard error of the mean (SEM) of three independent experiments. Scale bar: 50 μm.

To determine whether the fusion gene of *ACTB* and marker was successfully created by HR, ACTB-Cas9 and the donor vector containing 3xFLAG sequence were co-transfected into HeLa cells. Seventy-two hours after transfection, cell lysates were prepared, and expression of the fusion gene of *ACTB* and 3xFLAG (β-actin::3xFLAG) was analyzed by Western blotting (WB) (Fig. 2a). By immunoprecipitation (IP), we confirmed that signal detected by the anti-FLAG antibody represented β-actin::3xFLAG. Protein immunoprecipitated using the anti-FLAG antibody reacted to the anti-β-actin antibody, and no significant non-specific bands were observed (data not shown). Therefore, we concluded that the off-target effect of wild-type Cas9 did not affect the detection of β-actin::3xFLAG. Expression of β-actin::3xFLAG was also detected and confirmed by IP in HEK-293T cells (Fig. 2b) and detected in U-2 OS cells (data not shown). When we replaced the target gene with an arbitrarily chosen gene, *Receptor for activated C kinase 1 (RACK1)*, we successfully detected RACK1::3xFLAG in HeLa cells (Fig. 2c).

Next, to apply this strategy to the FC format, we replaced the marker with mClover. HeLa cells transfected with donor vector containing the mClover sequence and ACTB-Cas9 contained a significantly higher proportion of ACTB::mClover-positive cells than cells transfected with the donor vector and scr-Cas9 (Fig. 2d,e).

### Optimization of the assay system for the WB format

Next, we optimized the assay system in HeLa cells. First, we determined the optimal molar ratio of gRNA/Cas9 vector to donor vector. As shown in Supplementary Fig. S1a, molar ratios from 1:20 to 1:2 resulted in strong signals. We then optimized the amount of DNA using a gRNA/Cas9:donor ratio of 1:2, and obtained the most intense signal when 1.0 μg of total DNA was transfected into cells in a 3.5 cm dish (Supplementary Fig. S1b). Last, we optimized the optimal incubation time after transfection. β-actin::3xFLAG was detectable 48 hours after transfection and peaked 72 hours after transfection (Supplementary Fig. S1c). Based on all of these findings, we determined that the following conditions were optimal for the WB format: for HeLa cells in a 3.5 cm dish, harvest 72 hours after transfection using 1.0 µg DNA (0.5 µg each of gRNA/Cas9 vector and donor vector, corresponding to a molar ratio of 1:2).

### Assessment of HR deficiency due to derangement of the HR factors BRCA1 and BARD1

**Figure 3:**
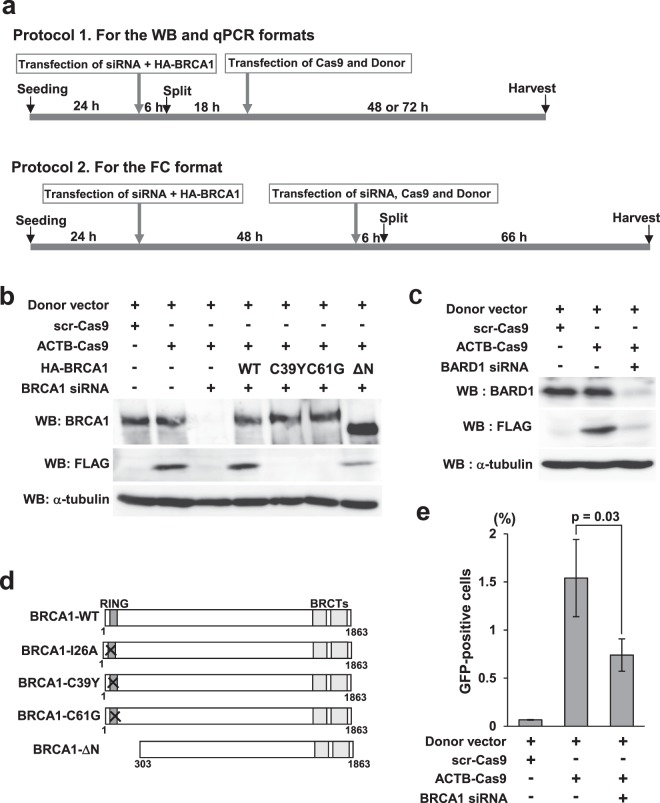
Validation of the assay system using knockdown of BRCA1 or BARD1. (**a**) Transfection protocols for each assay format. (**b**) Effect of BRCA1 knockdown and rescue on the expression of β-actin::3xFLAG. siRNA against the 3′-UTR of BRCA1 was transfected with the expression vectors for HA-BRCA1-WT, C39Y, C61G, and ΔN or an empty vector at the first transfection (Protocol 1). Cells were harvested for WB 72 hours and for pPCR 48 or 72 hours after transfection of ACTB-Cas9 and donor vectors. (**c**) Effect of BARD1 knockdown on the expression of β-actin::3xFLAG. siRNA against BARD1 was introduced in the first transfection (Protocol 1). Cells were harvested for WB 72 hours after transfection of ACTB-Cas9 and donor vectors. (**d**) Schematic illustration of BRCA1 variants used in the experiments. (**e**) Effect of BRCA1 knockdown on the expression of β-actin::mClover. siRNA against the 3′-UTR of BRCA1 was transfected at the first transfection (Protocol 2). At the second transfection, siRNA was transfected with ACTB-Cas9 and donor vectors. Cells were analyzed by FC 72 hours after the second transfection. Error bars indicate ± SEM of three independent experiments.

BRCA1 protein functions in HR by forming a heterodimer with BRCA1-associated RING domain protein (BARD1)^6^. To determine whether this assay could detect HR deficiency, we knocked down BRCA1 or BARD1 by RNAi and analyzed β-actin::3xFLAG expression by WB (Fig. 3a, Protocol 1). Knockdown of BRCA1 markedly decreased expression of β-actin::3xFLAG (Fig. 3b). Introduction of vector expressing HA-tagged wild-type BRCA1 (HA-BRCA1-WT) along with siRNA of BRCA1 rescued the expression of β-actin::3xFLAG to the control level (Fig. 3b). Knockdown of BARD1 also decreased the expression of β-actin::3xFLAG (Fig. 3c).

Multiple variants of BRCA1 are deficient in HR function^22,28,29,34–36^. We applied our method to testing the HR activities of two BRCA1 missense variants, C39Y and C61G, and an N-terminal deletion mutant (BRCA1-ΔN) that lacks the RING domain responsible for interaction with BARD1 (Fig. 3d). C39Y and C61G did not rescue expression of β-actin::3xFLAG in BRCA1-knockdown cells, whereas BRCA1-ΔN had partial rescue activity (Fig. 3b). These results are consistent with previous results obtained with the DR-GFP assay^22,28,29^.

Next, we analyzed the effect of BRCA1 knockdown by the FC format. In contrast to the WB format, in the FC format we observed no reduction in the proportion of ACTB::mClover-positive cells upon knockdown of BRCA1 when we used Protocol 1 in Fig. 3a (data not shown). When we introduced siRNA twice, as in Protocol 2 in Fig. 3a, the proportion of ACTB::mClover-positive cells was reduced (Fig. 3e). The FC format detected partial rescue of BRCA1-ΔN similarly to the WB format (data not shown).

### Genomic quantitative PCR (qPCR) enabled direct quantitation of HR activity

Although our assay could evaluate HR activity in cells such as HeLa, using either the WB or FC format, several challenges remained. First, because both formats depend on the expression of fusion proteins, the expression efficiency and stability of the fusion proteins could affect the results. Second, these formats are semi-quantitative and relatively insensitive. Consequently, they might be insufficient to detect very low levels of fusion gene products, making it difficult to detect subtle changes in HR. Third, these formats cannot evaluate HR activities in un-transcribed regions.

**Figure 4:**
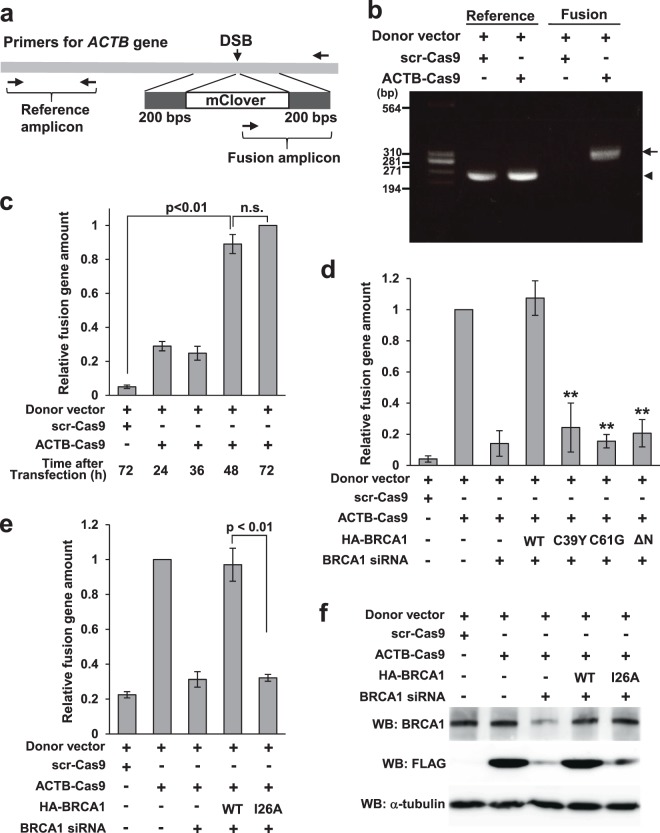
Quantitative PCR format for the new assay system. (**a**) Schematic illustration of knock-in and primer design. The donor vector contains 5′ and 3′ arms 200 bp in length and mClover as a marker. For amplification of the fusion gene, the forward primer is placed in mClover and the reverse primer in *ACTB*, outside the homologous sequence of the 3′ arm. A primer pair placed upstream of the DSB site is used as a reference. (**b**) Electrophoresis image of semi-quantitative PCR. Semi-quantitative PCR revealed specific amplification of the fusion gene in cells transfected with ACTB-Cas9 and donor vectors. Genomic DNA was extracted 72 hours after transfection of ACTB-Cas9 and donor vectors. Arrow or arrowhead indicates the fusion or reference amplicon, respectively. (**c**) Detection of the fusion gene by quantitative PCR. Genomic DNA was extracted 24, 36, 48, and 72 hours after transfection of ACTB-Cas9 and the donor vectors. Relative quantity of the fusion gene was calculated using the 72 hours sample as a control. Error bars indicate ± SEM of three independent experiments. n.s., not significant. (**d**) Quantitation of HR activities of BRCA1 variants using the qPCR format. Genomic DNA was extracted 72 hours after transfection of ACTB-Cas9 and donor vector (Fig. 3a, Protocol 1). Relative quantity of the fusion gene was calculated using a sample transfected with control siRNA and empty vector as a control. Error bars indicate ± SEM of three independent experiments. Significant differences relative to BRCA1-WT are indicated by asterisks. **p < 0.01. (**e**) Quantitation of HR activity of BRCA1-I26A variant using the qPCR format. The experiment was performed as in (**d**). Error bars indicate ± SEM of three independent experiments. (**f**) Analysis of HR activity of BRCA1-I26A variant in the WB format. siRNA and plasmids were transfected according to the protocol 1 in Fig. 3a and harvested 72 hours after the second transfection.

To overcome these problems, we developed a qPCR-based format for the assay. In this method, a plasmid containing mClover flanked by 200 bp arms was used as the donor vector (Fig. 4a). To specifically amplify *ACTB* fused with mClover, we designed a forward primer in mClover and a reverse primer 300 bp downstream from the stop codon of *ACTB*. A primer set around the start codon of *ACTB* was used as an internal control. In HeLa cells transfected with ACTB-Cas9 and the donor plasmid, we observed specific amplification of ACTB::mClover (Fig. 4b), and could specifically quantify the knock-in products by genomic qPCR (Fig. 4c). Moreover, incubation for 48 hours after transfection was sufficient for detection using the qPCR format, shorter than the 72 hours required for the WB format.

Next, we assessed the accuracy of the qPCR format using BRCA1 knockdown and rescue. BRCA1 knockdown significantly decreased expression of ACTB::mClover, and as in the previous experiments, exogenous expression of wild-type BRCA1, but not C39Y and C61G, rescued creation of ACTB::mClover (Fig. 4d). However, BRCA1-ΔN had no rescue activity in the qPCR format, despite the fact that it exhibited partial rescue activity in the WB and FC formats (Fig. 3b and data not shown) and DR-GFP assay^29^. These observations indicate that results obtained using the qPCR format might differ from those acquired using the WB format or DR-GFP assay.

We hypothesized that the qPCR format might be able to detect HR deficiency of some BRCA1 variants that were judged as HR-proficient or marginally proficient by the WB format or DR-GFP assay. The BRCA1-I26A variant (Fig. 3d), which is deficient in E3 ligase activity^41^, causes high sensitivity to cisplatin and olaparib^22^. However, BRCA1-I26A has been categorized as HR-proficient by DR-GFP assay^22,41^. When we measured the HR activity of BRCA1-I26A by the qPCR format, I26A variant did not show rescue activity (Fig. 4e). By contrast, the WB format revealed that I26A possessed partial rescue activity (Fig. 4f).

### The qPCR format enables measurement of HR activity in un-transcribed regions

**Figure 5:**
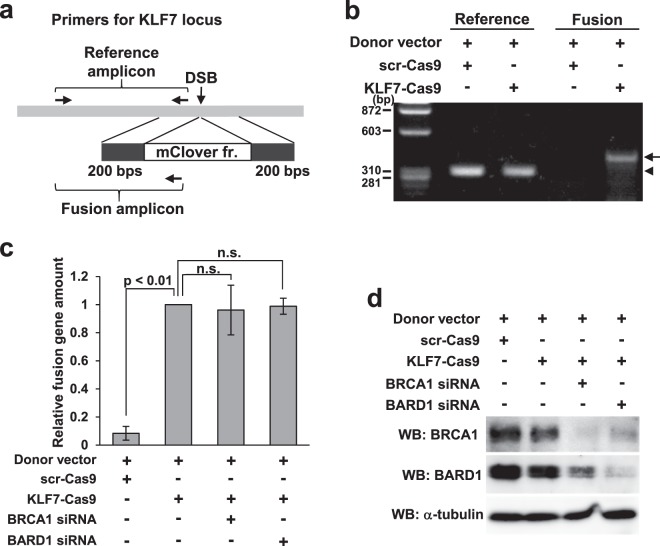
Detection of knock-in in a RAD51-unbound locus by qPCR. (**a**) Schematic illustration of knock-in and primer design. The donor vector contains 5′ and 3′ arms 200 bp in length and mClover as a marker. For amplification of the fusion gene, the forward primer is placed in mClover and the reverse primer in the *KLF7* locus, outside the homologous sequence of the 3′ arm. A forward primer placed in the 3′ homology sequence and the reverse primer used for the fusion amplicon were used for reference. (**b**) Electrophoresis image of semi-quantitative PCR. Genomic DNA was extracted 72 hours after transfection of KLF7-Cas9 and donor vectors. Arrow or arrowhead indicates the fusion or reference amplicon, respectively. (**c**) Quantitation of HR in the *KLF7* locus. Transfection was performed according to Protocol 1 in Fig. 3a. Genomic DNA was extracted 48 hours after the second transfection. Quantity of the fusion gene was calculated relative to a sample transfected with control siRNA. Error bars indicate ± SEM of three independent experiments. n.s., not significant. (**d**) WB analysis of samples prepared as in (**c**).

Aymard *et al*. reported that epigenetic modifications of histones control the distribution of DNA repair factors along DNA strands^40^, suggesting that HR activity might depend on the locus selected for measurement. Theoretically, the qPCR format could target any locus in the genome, irrespective of its transcriptional activity. Therefore, we attempted to quantify HR activity and evaluate the function of BRCA1 in a *KLF7-*neighboring locus (described simply as the *KLF7* locus), which is located in an extragenic region and is not accumulated by RAD51 after DSB generation^40^. To this end, we constructed a vector for the expression of gRNA/Cas9 and a donor vector for the *KLF7* locus, and then quantified the knock-in products by qPCR (Fig. 5a). We successfully detected and quantified the knock-in products in the *KLF7* locus (Fig. 5b,c). To determine the effect of BRCA1 knockdown on HR at the *KLF7* locus, we quantified the knock-in products after BRCA1 or BARD1 knockdown. Neither knockdown significantly decreased HR activity at the *KLF7* locus (Fig. 5c). Knockdown of BRCA1 and BARD1 were confirmed by WB (Fig. 5d).

### The contribution of BRCA1 and RAD51 to knock-in differs between loci

**Figure 6:**
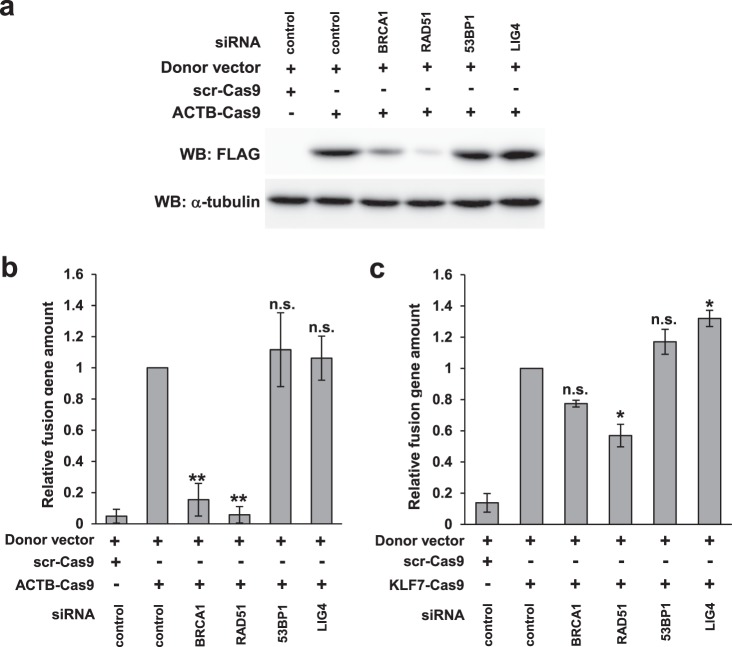
Dependence of knock-in on HR or NHEJ factors. (**a**) Effects of knocking down RAD51, 53BP1 or LIG4 on the expression of β-actin::3xFLAG. Transfection was performed according to Protocol 1 in Fig. 3a. (**b**,**c**) qPCR analysis of knock-in at the *ACTB* (**b**) or *KLF7* (**c**) locus in RAD51, 53BP1, or LIG4 knock-down cells. Genomic DNA was extracted 48 h after transfection of ACTB-Cas9 and a donor vector (Fig. 3a, Protocol 1). The amount of the fusion gene was calculated relative to a sample transfected with control siRNA. Error bars indicate ± SEM of three independent experiments. Significant differences relative to the sample transfected with control siRNA are indicated by asterisks. *p < 0.05, **p < 0.01, n.s., not significant.

To analyze the contribution of the HR and NHEJ pathways to knock-in of marker sequences, we knocked down HR- or NHEJ-related factors and quantified knock-in products using the WB or qPCR formats. As shown in Fig. 6a, expression of β-actin::3xFLAG fell significantly after knockdown of RAD51 and BRCA1. By contrast, knockdown of NHEJ-related factors 53BP1 or DNA ligase IV (LIG4) did not affect expression of β-actin::3xFLAG. Similar results were obtained by qPCR of the *ACTB* locus (Fig. 6b). Although knockdown of RAD51 also reduced the knock-in efficiency at the *KLF7* locus, the decrease was relatively small compared with that at the *ACTB* locus (Fig. 6c). Knockdown of RAD51, 53BP1, and LIG4 was confirmed by WB (Supplementary Fig. S2).

## Discussion

In this study, we developed an assay, Assay for Site-specific HR Activity (ASHRA), to evaluate HR activity at endogenous gene loci by transient transfection.

First, we attempted to detect products of a fusion gene created by HR-mediated knock-in of marker sequences into endogenous target genes. The CRISPR/Cas9 system was used to create DSBs at specific sites. Expression of a fusion gene of β-actin and 3xFLAG was successfully detected by WB after transient transfection of the gRNA/Cas9 expression vector and the donor vector, without antibiotic selection. Expression of the fusion protein was confirmed by IP. This observation suggested that the off-target effect of wild-type Cas9 (i.e., rather than nickase) did not affect the detection of HR activity. We observed expression of fusion genes created by HR in several cell lines other than HeLa, as well as fusions created using another target gene. On the basis of our findings, we conclude that expression of the fusion gene can be detected independently of cell type and genomic locus.

We then optimized the amount of vector, the molar ratio of the gRNA/Cas9 expression vector to the donor vector, and the timing of harvest after transfection using HeLa cells. The optimal ratio of the gRNA/Cas9 expression vector to the donor vector ranged from 1:20 to 1:2. We decided to use a ratio of 1:2 in our experiments. The optimal amount of DNA was 0.5–1.0 μg for HeLa cells in a 3.5 cm dish, and usually did not induce severe cell damage after transfection. Expression of the fusion gene was observed faintly at 48 hours and strongly at 72 hours after transfection by WB. On the other hand, when using the qPCR format, the knock-in product itself could be clearly detected 48 hours after transfection. This difference might be due to the additional time required for translation of the mRNA transcribed from the fusion gene.

We observed a significant decrease in the level of the expression of fusion gene by the WB and FC and knock-in by the qPCR formats when either BRCA1, BARD1, or RAD51 was knocked down, suggesting that ASHRA was capable of detecting HR deficiency. However, there were some differences between the formats. For the WB and qPCR formats, single transfection of siRNA was sufficient for detection of HR deficiency due to knockdown of BRCA1, whereas the FC format required repeated transfections of siRNA. Like the FC format of ASHRA, the DR-GFP assay also required repeated transfection of siRNA of BRCA1 to detect HR deficiency^29^. In FC, cells are recognized as negative for mClover only when knock-in of mClover fails at all alleles. Therefore, more thorough knockdown of BRCA1 or BARD1 might be required for the DR-GFP assay and the FC format of ASHRA than for the WB and qPCR formats of ASHRA.

Recently, Anantha *et al*. reported the functions of a number of BRCA1 variants in HR activity and resistance to cisplatin and the PARP inhibitor olaparib^22^. When they analyzed HR activity by DR-GFP assay, they found that BRCA1-C61G was completely defective in HR, whereas BRCA1-Δ1-143, which is similar to BRCA1-ΔN used in the experiments described here, was partially functional. These data are consistent with our previous results obtained with the DR-GFP assay^29^ and the WB and FC formats of ASHRA. Despite the residual HR activity, BRCA1-Δ1-143 confers high sensitivity to cisplatin and olaparib. Interestingly, several missense variants, including I26A, also exhibited a discrepancy between HR activity and chemosensitivity. By contrast, the qPCR format of ASHRA revealed that both the BRCA1-ΔN and -I26A variants were completely defective in HR, a finding consistent with chemosensitivity. These data indicate that the qPCR format of ASHRA is suitable for evaluating the HR activity of BRCA1 variants, and for predicting their sensitivity to therapies that induce DNA damage (e.g., DSBs and DNA crosslinks) and to PARP inhibitors (Table 1).

**Table 1:** Summary of HR activity, as determined by ASHRA or DR-GFP assay, and sensitivity to chemotherapy or radiotherapy.

BRCA1	HR by ASHRA		HR by DR-GFP^*^	Sensitivity	
	WB	qPCR		Chemotherapy^§^	Radiotherapy^ǂ^
WT	As wild type	As wild type	As wild type	Resistant	Resistant
I26A	Intermediate	Deleterious	Intermediate	Sensitive	Sensitive
C39Y	Deleterious	Deleterious	Deleterious	Sensitive	Sensitive
C61G	Deleterious	Deleterious	Deleterious	Sensitive	Sensitive
N-terminal deletant	Intermediate	Deleterious	Intermediate	Sensitive	ND

For evaluation of N-terminal deletion of BRCA1, ΔN was used in ASHRA, ΔN and Δ1–134 in the DR-GFP assay, and Δ1–134 in the chemosensitivity assay. *From ref.^22,29,35^; ^§^From 29 and 35; ^ǂ^From 25. ND, not determined.

Although the I26A variant was completely defective in the qPCR format and causes high sensitivity to cisplatin and olaparib, the variant is resistant to mitomycin C^41^. In addition, mice harboring the I26A variant can survive but develop cancers^41,42^. I26A variant is deficient in E3 ubiquitin ligase activity, but proficient in binding to BARD1^43,44^. By contrast, C61G variant loses both activities of E3 ubiquitin ligase and binding to BARD1^45,46^. Mice having C61G variant is embryonic lethal^47^. These suggest that BRCA1/BARD1 heterodimerization is required for survival of mice. BRCA1 has many interaction partners that contribute various cellular functions. Each assay (including each format of the ASHRA and DR-GFP assays and the cell survival assay against chemotherapeutics) reflects only one or several aspects of BRCA1 function. The phenotypes of the genetically modified mice are affected by various BRCA1 functions. The qPCR format of ASHRA might evaluate activity of the core machinery responsible for HR. The data suggest that activity of BRCA1 in HR might be not essential for survival and resistance to some DNA damaging agents.

It remains unclear why some residual HR activity was detected in certain BRCA1 variants, including ΔN and I26A, in the WB or FC formats of ASHRA and by the DR-GFP assay (which are dependent on expression of HR products) in contrast to the qPCR format of ASHRA. Like the C61G variant^45^, both the ΔN and I26A variants are deficient in E3 ubiquitin ligase activity. Therefore, these BRCA1 mutants might affect protein turnover and other cellular activities. In addition, BRCA1 also regulates transcription and chromatin remodeling. These activities of BRCA1 variant could affect expression of HR products. However, the results obtained from the qPCR format of ASHRA should reflect direct HR activity on the DNA level and therefore not be influenced by the activities of protein turnover or expression. Moreover, the donor vectors for the WB and FC formats contain about 1500 bp of *ACTB* sequences in 5′ and 3′ of marker genes, respectively, whereas the donor vector for qPCR format has only 200 bp of *ACTB* sequences in 5′ and 3′of mClover gene. These might affect the efficiency of HR and cause the discrepancy of the results among the formats ASHRA. Expression levels of BRCA1 variants might affect the results. For example, expression of ΔN is higher than BRCA1-WT, potentially affecting the results. Furthermore, the DR-GFP assay uses two GFP cassettes located very close together on a single chromosome. In ASHRA, the DSB and the donor sequence exist on separate molecules. These differences may be responsible for the discrepancies in the results obtained by the DR-GFP assay and ASHRA.

In addition, the qPCR format of ASHRA can measure HR activity in non-expressing loci in the genome, which was previously impossible because the conventional assays depend on the expression of HR products. Therefore, our assay system will enable the investigation of HR pathways in transcriptionally inactive regions. Interestingly, we found that neither knockdown of BRCA1 nor BARD1 significantly affected HR activity at the *KLF7* locus. Although knockdown of RAD51 decreased HR activity at the *ACTB* and *KLF7* loci, the decrease was relatively small at the *KLF7* locus. Knockdown of NHEJ-related factors, 53BP1 and LIG4, did not suppress HR activity at the *ACTB* and *KLF7* loci. The *KLF7* locus is located in an extragenic region and is reported by Aymard *et al*. to be an RAD51-unbound locus^40^. These observations suggested that HR at the *KLF7* locus might not require the BRCA1/BARD1 heterodimer, and that some BRCA1/BARD1-independent pathway may be involved in HR. RAD51 might contribute at least in part to HR at the *KLF7* locus. What factors contribute to the HR activity in the *KLF7* locus remains to be elucidated.

HR activity quantified by ASHRA is not directly comparable between different loci in this study. The amount of the fusion gene determined by the qPCR format was higher at the *ACTB* locus than at the *KLF7* locus (data not shown). The efficiency of DSB creation by Cas9 and the resultant amount of fusion gene created could depend on chromatin structure. Therefore, to compare HR activities in different loci, normalization by efficiencies of transfection and DSB creation will be required.

In this study, HR activity at the *ACTB* locus was better correlated to chemosensitivity than activity at the *KLF7* locus. This raises the important question of which region’s HR activity best correlates with chemosensitivity. We have not comprehensively investigated the correlation of HR activity in multiple loci measured by ASHRA with sensitivity to chemotherapy or radiotherapy. Therefore, in the future studies, we could determine the best locus for evaluation of HR to most accurately predict sensitivity to chemotherapy or radiotherapy. In addition, we have not yet applied this assay to measurement of HR activity in clinical specimens. These goals should be pursued in future studies.

In conclusion, we developed ASHRA to evaluate HR activity at any genomic region in a cell of interest. ASHRA is simple, cost-effective, and rapid, and can be performed in flexible formats. The qPCR format of ASHRA is especially advantageous in terms of its quantitative nature, sensitivity, and accuracy. Direct evaluation of HR deficiency by ASHRA will be useful for accurately stratifying cancer patients who are likely to benefit from treatments with DNA-damaging agents and chemotherapy using PARP inhibitors. We anticipate that ASHRA will contribute to drug development, investigation of the HR machinery, and patient stratification.

## Methods

### Cell lines and culture

HEK-293T and HeLa cells were purchased from American Type Culture Collection (Manassas, VA, USA). U-2 OS cells were provided by the Cell Resource Center of Institute of Development, Aging, and Cancer, Tohoku University (Sendai, Japan). HEK-293T, HeLa, and U-2 OS cells were maintained in Dulbecco’s modified Eagle’s medium (DMEM) supplemented with 8% fetal bovine serum (FBS). All cells were incubated in an atmosphere containing 5% CO_2_.

### Construction and transfection of plasmids

pBluescript SK II+ was purchased from Stratagene (San Diego, CA, USA). LentiCRISPR v2 plasmid was purchased from Addgene (Cambridge, MA, USA, #52961). LentiCRISPR plasmids expressing gRNA against *ACTB*, *RACK1*, and *KLF7* or scrambled gRNA were constructed according to the depositor’s instructions. Target sequences of gRNA against *ACTB*, *RACK1*, and *KLF7* were 5′-CCGCCTAGAAGCATTTGCGG-3′, 5′-AAACTTCTAGCGTGTGCCAA-3′, and 5′-ATCACTCCAGCTCGTGGATC-3′, respectively. The sequence of scrambled gRNA was 5′-CCTGGGTTAGAGCTACCGCA-3′. To construct HR donor vectors against *ACTB*, sequences from −1554 to +1527 or −200 to +200 relative to the stop codon of *ACTB* were amplified by PCR and cloned into pBluescript using InFusion Clonase (Takara Bio, Shiga, Japan). DNA fragments encoding 3xFLAG and mClover were synthesized and amplified by PCR from pMK290 plasmid (a gift from Dr. Masato Kanemaki, Div. of Molecular Cell Engineering, National Institute of Genetics, Mishima, Japan)^48^ respectively and cloned into HR donor vectors just 5′ of the stop codon of *ACTB* using InFusion Clonase or T4 DNA ligase (Takara Bio). To construct the HR donor vector for *RACK1*, sequences from −1337 to +1378 relative to the stop codon of *RACK1* were cloned in pBluescript, and the 3xFLAG sequence was inserted just 5′ of the stop codon. To construct the HR donor vector against the *KLF7* locus, sequences from −200 to +200 relative to the DSB site were amplified using primers containing the GFP primer sequence, and the amplicon was cloned into pBluescript with T4 DNA ligase. Polyethylenimine MAX (Polysciences, Warrington, PA, USA) was used for plasmid transfection.

### RNAi

siRNAs used for knockdown of *BRCA1* and *BARD1* were synthesized using the Silencer siRNA Construction Kit (Thermo-Fisher Scientific, Waltham, MO, USA). siRNAs used for knockdown of *RAD51*, *53BP1*, and *LIG4* were purchased from Integrated DNA technologies (Coralville, IA, USA). Target sequences of siRNAs were as follows: 5′-AAGGUUUCAAAGCGCCAGUCA-3′ for the 3′-UTR of BRCA1, 5′-GAGUAAAGCUUCAGUGCAATT-3′ for BARD1, 5′-GUCACAAACUGAUCUAAAAUGUUTA-3′ for RAD51, 5′-GCUUGAGUUCUCACAGAAUUGAUGA-3′ for 53BP1, and 5′-GAUACAGACUUGAACCAACUGAAGG-3′ for LIG4. The Silencer negative control siRNA template set was used as the control. Lipofectamine RNAiMAX (Thermo-Fisher Scientific) was used for transfection of siRNA. Trans-IT X2 (Mirus Bio, Madison, WI, USA) was used for co-transfection of siRNA and plasmid DNA.

### WB

Cells were lysed in 1× sample buffer (25 mM HEPES [pH 7.6], 150 mM NaCl, 1% Nonidet P-40, 2% SDS, 10% sucrose) supplemented with protease inhibitor cocktail (Roche, Basel, Switzerland) and phosphatase inhibitor cocktail (5 mM sodium fluoride, 200 μM sodium orthovanadate, 1 mM sodium molybdate, 2 mM sodium pyrophosphate, and 2 mM disodium β-glycerophosphate at final concentrations), yielding whole-cell lysate. SDS-PAGE and WB were performed as previously described^49^. Primary antibodies were as follows: anti-FLAG (1:5000, clone M2, Sigma-Aldrich, St. Louis, MO, USA), anti-β-actin (1:3000, C4, SantaCruz Biotechnology, Dallas, TX, USA), anti-GFP (1:2000, Wako), and anti-α-tubulin (1:5000, Merck, Darmstadt, Germany). Secondary antibodies were as follows: HRP-tagged anti-mouse IgG (GE Healthcare, Little Chalfont, UK), HRP-tagged anti-rabbit IgG (GE Healthcare), and HRP-tagged anti-native mouse IgG (TrueBlot, Rockland, Limerick, PA, USA). Signals were detected using ECL substrate (ATTO, Tokyo, Japan) on a CCD imager (Image Quant LAS 4000 mini, GE Healthcare).

### IP

Cells were lysed in lysis buffer (10 mM HEPES [pH 7.6], 250 mM NaCl, 0.5% Nonidet P-40, 5 mM EDTA) supplemented with protease inhibitor and phosphatase inhibitor cocktails, and then cleared by centrifugation at 2500 *g* for 10 minutes. The lysate was rotated with 2 μl of anti-FLAG antibody (clone M2) at 4 °C overnight. The lysate was rotated for another hour with Protein G–Sepharose (GE Healthcare). After vigorous washing, proteins were eluted in 1× sample buffer and analyzed by WB.

### FC

Cells were trypsinized and collected by centrifugation. Single-cell suspensions were prepared using DMEM without phenolphthalein. mClover-positive cells were counted on a Cytomics FC500 flow cytometer (Beckman-Coulter, Brea, CA, USA).

### Genomic quantitative and semi-quantitative PCR

Genomic DNA was extracted by the conventional method using glass beads. Quantitative PCR (qPCR) was performed using GoTaq qPCR master mix (Promega, Madison, WI, USA) on a CFX96 instrument (Bio-Rad, Hercules, CA, USA). Relative quantity of the knock-in allele was calculated by the ΔΔCt method using the following formulae: 2(^−ΔΔCt^), ΔΔCt = ΔCt[sample] − ΔCt[positive control], ΔCt = Ct[fusion allele] − Ct[reference allele]. Semi-quantitative PCR was performed using EmeraldAmp MAX (Takara Bio), and the products were run on agarose gels. Primer sequences are as follows: ACTB-ref-F (5′-GTCACCAACTGGGACGACAT-3′) and ACTB-ref-R (5′-AGAACCAGTGAGAAAGGGCG-3′) for the reference allele of *ACTB*; GFP-F1 (5′-GTCCTGCTGGAGTTCGTGACCG-3′) and ACTB-common-R1 (5′-GTGCAATCAAAGTCCTCGGC-3′) for the fusion allele of *ACTB*; KLF7-common-F1 (5′-AGCCGGTGTCGTGGACAAGT-3′) and KLF7-R1 (5′-CTGCACTGTACACGCTGGATG-3′) for the reference allele of *KLF7*; and KLF7-common-F1 and GFP-R1 (5′-CGGTCACGAACTCCAGCAGGAC-3′) for the fusion allele of *KLF7*.

### Image processing

ImageJ v1.49 (http://imagej.nih.gov/ij/) was used for image analysis.

### Statistical analysis

Statistical analysis was performed using the JMP 12 software (SAS Institute, Tokyo, Japan). Graphs were constructed using Excel 2016 (Microsoft Corporation, Redmond, WA, USA). Statistical comparisons between two different samples were made by two-tailed Welch’s test. Comparisons between more than two samples were made by ANOVA. When the result of ANOVA indicated a significant difference, post-hoc comparisons were made by Dunnett’s test to calculate p-values. A p-value of <0.05 was considered significant.

## Supplementary information

Supplementary information

## Data Availability

The data supporting the conclusions of this article are included within the article. The materials used in this article are available from the corresponding author.

## References

[1] Shaltiel IA, Krenning L, Bruinsma W, Medema RH (2015). The same, only different - DNA damage checkpoints and their reversal throughout the cell cycle. J. Cell Sci..

[2] Dasika GK (1999). DNA damage-induced cell cycle checkpoints and DNA strand break repair in development and tumorigenesis. Oncogene.

[3] Prakash R, Zhang Y, Feng W, Jasin M (2015). Homologous Recombination and Human Health: The Roles of BRCA1, BRCA2, and Associated Proteins. Cold Spring Harb. Perspect. Biol..

[4] Sawyer SL (2015). Biallelic mutations in BRCA1 cause a new Fanconi anemia subtype. Cancer Discov.

[5] Martins FC (2012). Evolutionary pathways in BRCA1-associated breast tumors. Cancer Discov.

[6] Huen MSY, Sy SMH, Chen J (2010). BRCA1 and its toolbox for the maintenance of genome integrity. Nat. Rev. Mol. Cell Biol..

[7] Holm C, Covey JM, Kerrigan D, Pommier Y (1989). Differential requirement of DNA replication for the cytotoxicity of DNA topoisomerase I and II inhibitors in Chinese hamster DC3F cells. Cancer Res..

[8] Ross W, Rowe T, Glisson B, Yalowich J, Liu L (1984). Role of topoisomerase II in mediating epipodophyllotoxin-induced DNA cleavage. Cancer Res..

[9] Ross WE, Glaubiger D, Kohn KW (1979). Qualitative and quantitative aspects of intercalator-induced DNA strand breaks. Biochim. Biophys. Acta.

[10] Kan C, Zhang J (2015). BRCA1 Mutation: A Predictive Marker for Radiation Therapy?. Int. J. Radiat. Oncol. Biol. Phys..

[11] Gerratana L, Fanotto V, Pelizzari G, Agostinetto E, Puglisi F (2016). Do platinum salts fit all triple negative breast cancers?. Cancer Treat. Rev..

[12] Helleday T (2011). The underlying mechanism for the PARP and BRCA synthetic lethality: Clearing up the misunderstandings. Mol. Oncol.

[13] Audeh MW (2010). Oral poly(ADP-ribose) polymerase inhibitor olaparib in patients with BRCA1 or BRCA2 mutations and recurrent ovarian cancer: A proof-of-concept trial. Lancet.

[14] Sandhu SK (2013). The poly(ADP-ribose) polymerase inhibitor niraparib (MK4827) in BRCA mutation carriers and patients with sporadic cancer: A phase 1 dose-escalation trial. Lancet Oncol..

[15] Drew Y (2011). Therapeutic potential of poly(ADP-ribose) polymerase inhibitor AG014699 in human cancers with mutated or methylated BRCA1 or BRCA2. J. Natl. Cancer Inst.

[16] Kaufman B (2015). Olaparib monotherapy in patients with advanced cancer and a germline BRCA1/2 mutation. J. Clin. Oncol..

[17] Stefansson OA (2009). Genomic profiling of breast tumours in relation to BRCA abnormalities and phenotypes. Breast Cancer Res..

[18] Nik-Zainal S (2016). Landscape of somatic mutations in 560 breast cancer whole-genome sequences. Nature.

[19] Kanchi KL (2014). Integrated analysis of germline and somatic variants in ovarian cancer. Nat. Commun..

[20] Maxwell KN (2017). BRCA locus-specific loss of heterozygosity in germline BRCA1 and BRCA2 carriers. Nat. Commun..

[21] Denkert C, Liedtke C, Tutt A, von Minckwitz G (2017). Molecular alterations in triple-negative breast cancer—the road to new treatment strategies. Lancet.

[22] Anantha RW (2017). Functional and mutational landscapes of BRCA1 for homology-directed repair and therapy resistance. Elife.

[23] Byrski T (2009). Response to neoadjuvant therapy with cisplatin in BRCA1-positive breast cancer patients. Breast Cancer Res. Treat..

[24] Scully R (1999). Genetic analysis of BRCA1 function in a defined tumor cell line. Mol. Cell.

[25] Ruffner H, Joazeiro CA, Hemmati D, Hunter T, Verma IM (2001). Cancer-predisposing mutations within the RING domain of BRCA1: loss of ubiquitin protein ligase activity and protection from radiation hypersensitivity. Proc. Natl. Acad. Sci. USA.

[26] Swisher EM (2008). Secondary BRCA1 mutations in BRCA1-mutated ovarian carcinomas with platinum resistance. Cancer Res..

[27] Husain A, He G, Venkatraman ES, Spriggs DR (1998). BRCA1 up-regulation is associated with repairmediated resistance to cis-diamminedichloroplatinum(II). Cancer Res..

[28] Towler WI (2013). Analysis of BRCA1 variants in double-strand break repair by homologous recombination and single-strand annealing. Hum. Mutat..

[29] Ransburgh DJR, Chiba N, Ishioka C, Toland AE, Parvin JD (2010). Identification of breast tumor mutations in BRCA1 that abolish its function in homologous DNA recombination. Cancer Res..

[30] Kandoth C (2013). Mutational landscape and significance across 12 major cancer types. Nature.

[31] Watkins JA, Irshad S, Grigoriadis A, Tutt ANJ (2014). Genomic scars as biomarkers of homologous recombination deficiency and drug response in breast and ovarian cancers. Breast Cancer Res..

[32] Nakanishi K, Cavallo F, Brunet E, Jasin M (2011). Homologous recombination assay for interstrand crosslink repair. Methods Mol. Biol..

[33] Seluanov, A., Mao, Z. & Gorbunova, V. Analysis of DNA double-strand break (DSB) repair in mammalian cells. *J. Vis. Exp*. **43**, 10.3791/2002 (2010). 10.3791/2002 PMC3157866 20864925

[34] Starita LM (2015). Massively parallel functional analysis of BRCA1 RING domain variants. Genetics.

[35] Bouwman P (2013). A high-throughput functional complementation assay for classification of BRCA1 missense variants. Cancer Discov.

[36] Lu C (2015). Patterns and functional implications of rare germline variants across 12 cancer types. Nat. Commun..

[37] Lips EH (2017). BRCA1-mutated estrogen receptor positive breast cancer shows BRCAness, suggesting sensitivity to drugs targeting homologous recombination deficiency. Clin. Cancer Res..

[38] Pujade-Lauraine E (2017). Olaparib tablets as maintenance therapy in patients with platinum-sensitive, relapsed ovarian cancer and a BRCA1/2 mutation (SOLO2/ENGOT-Ov21): a double-blind, randomised, placebo-controlled, phase 3 trial. Lancet Oncol..

[39] Pinder J, Salsman J, Dellaire G (2015). Nuclear domain ‘knock-in’ screen for the evaluation and identification of small molecule enhancers of CRISPR-based genome editing. Nucleic Acids Res..

[40] Aymard F (2014). Transcriptionally active chromatin recruits homologous recombination at DNA double-strand breaks. Nat. Struct. Mol. Biol..

[41] Reid LJ (2008). E3 ligase activity of BRCA1 is not essential for mammalian cell viability or homologydirected repair of double-strand DNA breaks. Proc. Natl. Acad. Sci. USA.

[42] Shakya R (2011). BRCA1 tumor suppression depends on BRCT phosphoprotein binding, but not its E3 ligase activity. Science.

[43] Brzovic PS (2003). Binding and recognition in the assembly of an active BRCA1/BARD1 ubiquitin-ligase complex. Proc. Natl. Acad. Sci. USA.

[44] Christensen DE, Brzovic PS, Klevit RE (2007). E2-BRCA1 RING interactions dictate synthesis of monoor specific polyubiquitin chain linkages. Nat. Struct. Mol. Biol..

[45] Hashizume R (2001). The RING heterodimer BRCA1-BARD1 is a ubiquitin ligase inactivated by a breast cancer-derived mutation. J. Biol. Chem..

[46] Wu LC (1996). Identification of a RING protein that can interact in vivo with the BRCA1 gene product. Nat. Genet..

[47] Drost R (2011). BRCA1 RING function is essential for tumor suppression but dispensable for therapy resistance. Cancer Cell.

[48] Natsume T, Kiyomitsu T, Saga Y, Kanemaki MT (2016). Rapid Protein Depletion in Human Cells by Auxin-Inducible Degron Tagging with Short Homology Donors. Cell Rep..

[49] Matsuzawa A (2014). The BRCA1/BARD1-interacting protein OLA1 functions in centrosome regulation. Mol. Cell.

