# Supplementary material for: Evaluation of site-specific homologous recombination activity of BRCA1 by direct quantitation of gene editing efficiency
Source: Sci Rep. 2019 Feb 7;9:1644. doi: 10.1038/s41598-018-38311-x (PMC6367331; doi:10.1038/s41598-018-38311-x)
Supplement: Supplementary file 1 — Supplementary information [file 41598_2018_38311_MOESM1_ESM.pdf]

Evaluation of site-specific homologous recombination activity of BRCA1 by direct quantitation of gene editing efficiency

Yuki Yoshino[1, §], Shino Endo[1, §], Zhenghao Chen [1], Huicheng Qi[1], Gou Watanabe[2], Natsuko Chiba[1, *]

[1] Department of Cancer Biology, Institute of Aging, Development, and Cancer, Tohoku University, 4-1 Seiryo-machi, Aoba-ku, Sendai 980-8575, Japan
[2] Tohoku Medical and Pharmaceutical University, Sendai 983-8512, Japan
§ These authors contributed equally to this work.
* To whom correspondence should be addressed. Tel: +81-22-717-8477; Fax: +81-22-717-8482; Email: natsuko.chiba.c7@tohoku.ac.jp

Supplementary Figure S1

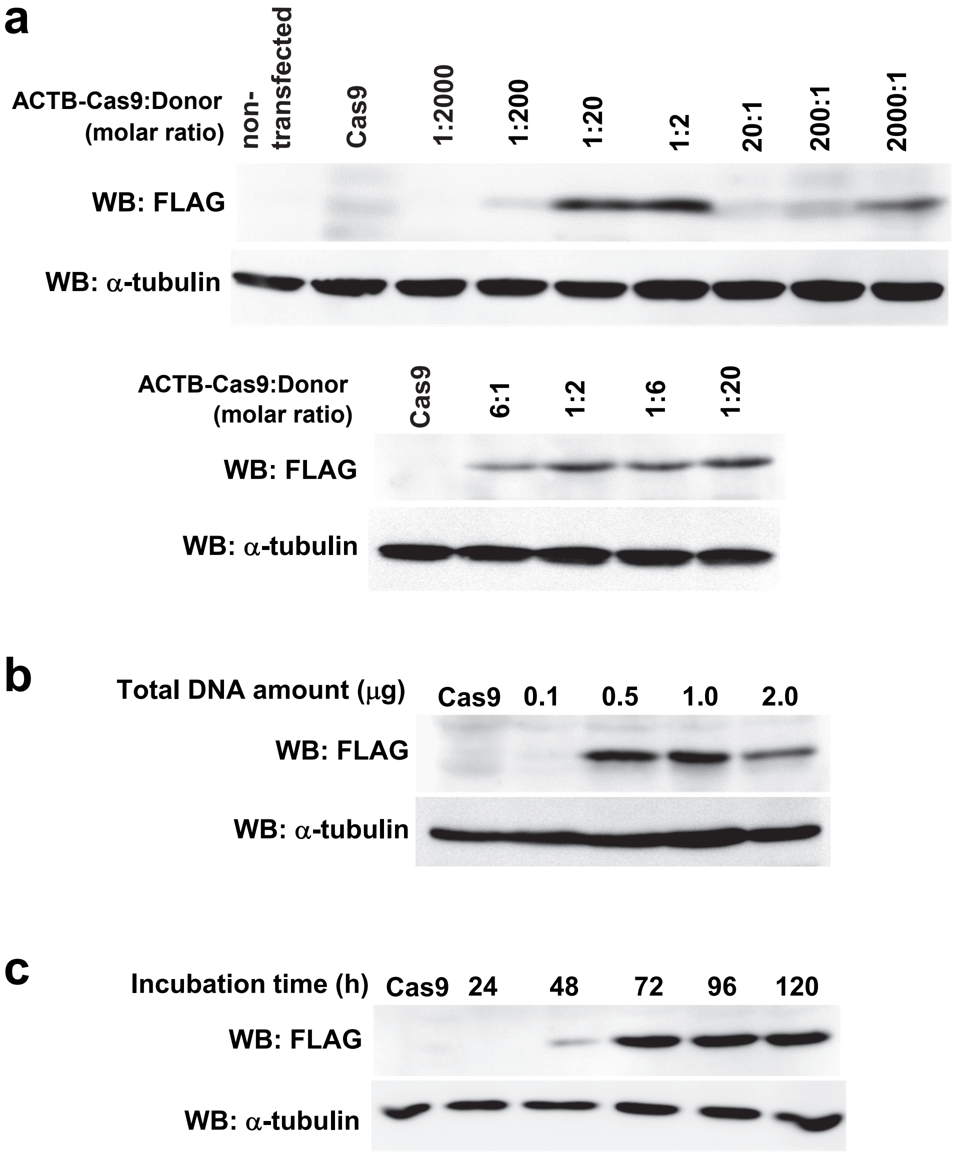

Supplementary Figure S1.

Optimization of the assay system for HeLa cells. (a) Optimization of ACTB-Cas9/donor vector ratio. ACTB-Cas9 and the donor vectors (total 1.0 μg) were transfected into HeLa cells in various molar ratios. Cells were harvested for WB 72 hours after transfection. no TF, no transfection. Cas9 means cells harvested 72 hours after transfection using 1.0 μg DNA (0.5 μg each of Cas9 vector (no gRNA) and donor vector). (b) Optimization of total DNA amount. Various amounts of total DNA were transfected, with a fixed molar ratio of ACTB-Cas9/donor vector (1:2). Cells were harvested for WB 72 hours after transfection. Cas9 means cells harvested 72 hours after transfection using 1.0 μg DNA (0.5 μg each of Cas9 vector and donor vector). (c) Effects of incubation time after transfection. A total of 1.0 μg of plasmid (1:2 ratio of ACTB-Cas9:donor vector) was transfected. Cells were harvested for WB 24, 48, 72, 96, and 120 hours after transfection. Cas9 means cells harvested 72 hours after transfection using 1.0 μg DNA (0.5 μg each of Cas9 vector and donor vector).

Supplementary Figure S2

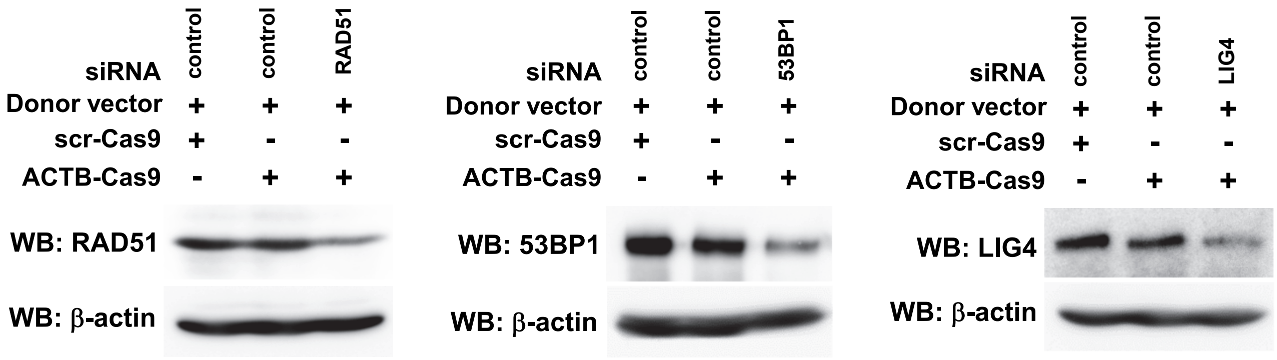

Supplementary Figure S2.

WB analysis of samples prepared as in Fig. 6b. Knockdown of RAD51, 53BP1, and LIG4 were confirmed.

Supplementary Figure S3

Fig. 2a

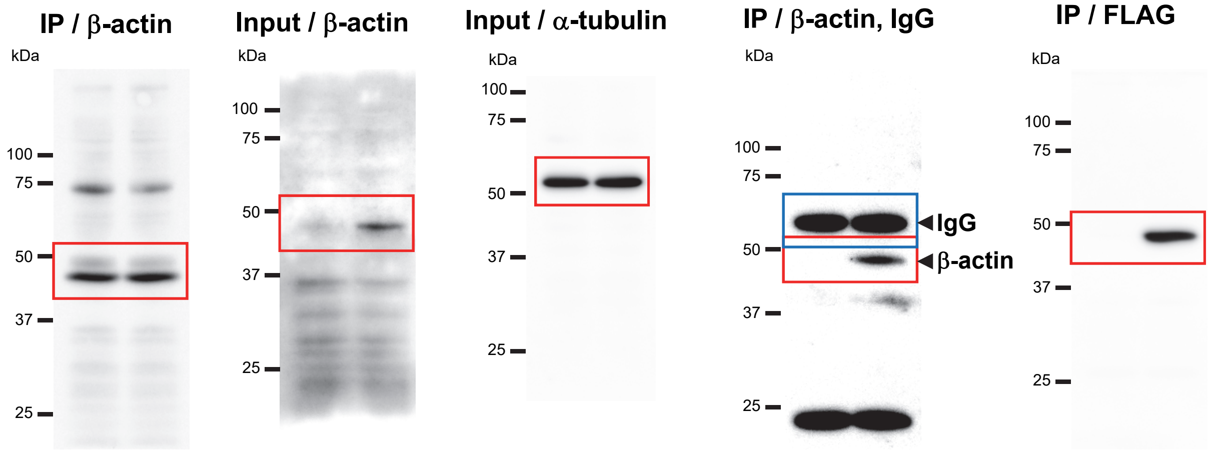

Fig. 2b

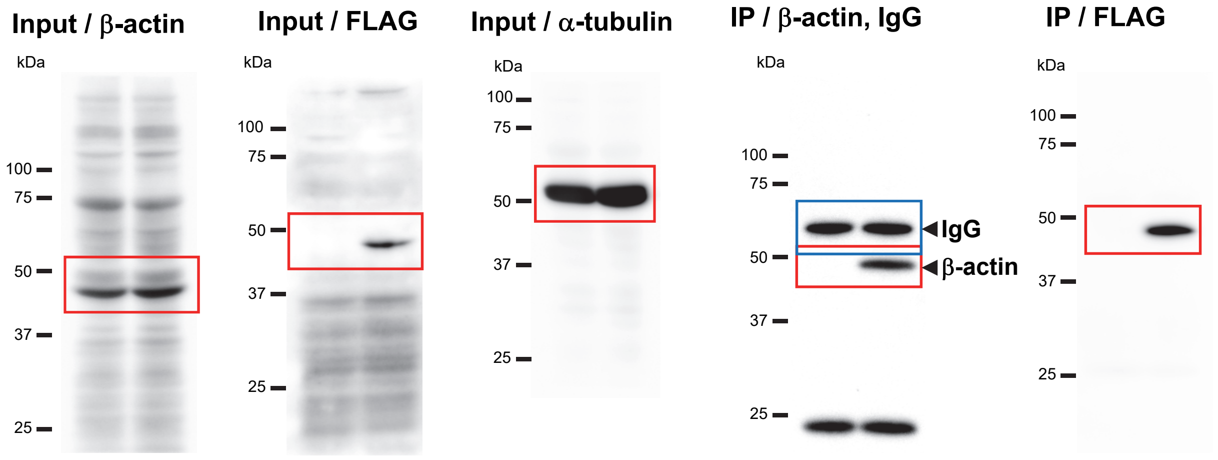

Fig. 2c

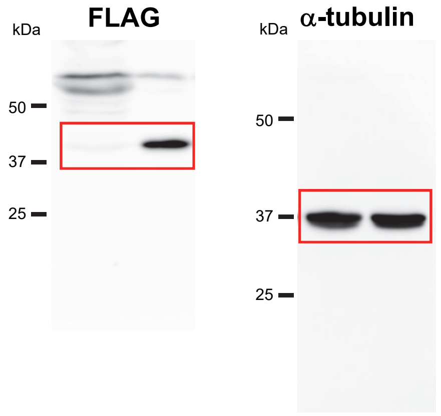

Supplementary Figure S3.
Uncropped images of Western blotting of Fig. 2a, b, and c.

Supplementary Figure S4

Fig. 3b

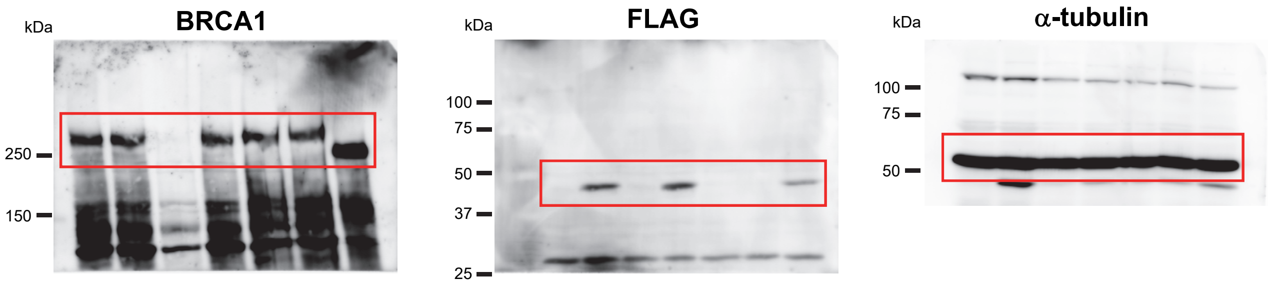

Fig. 3c

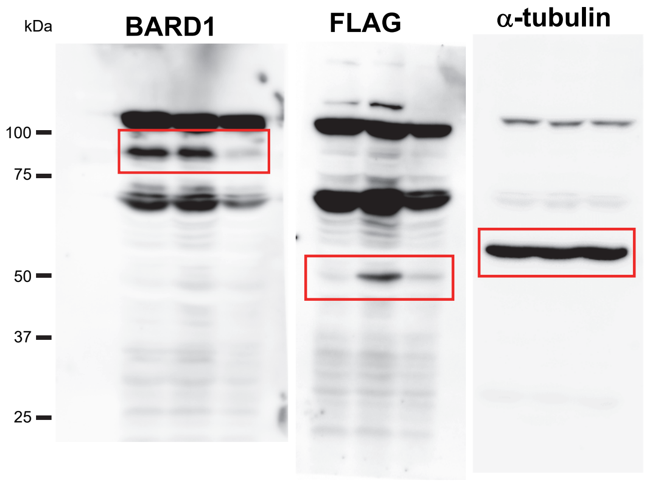

Supplementary Figure S4.
Uncropped images of Western blotting of Fig. 3b and c.

Supplementary Figure S5

Fig. 4f

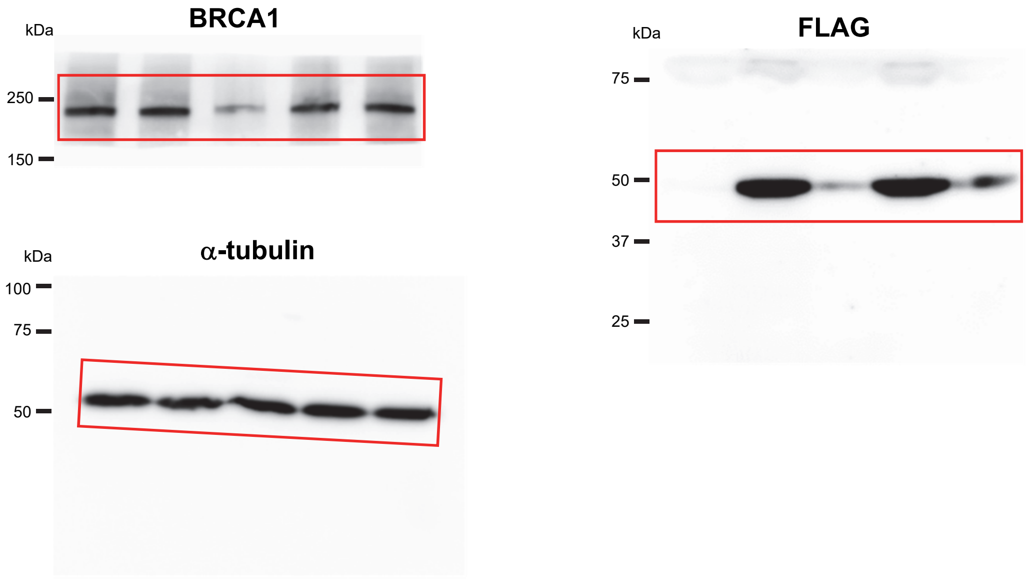

Supplementary Figure S5.
Uncropped images of Western blotting of Fig. 4f.

Supplementary Figure S6

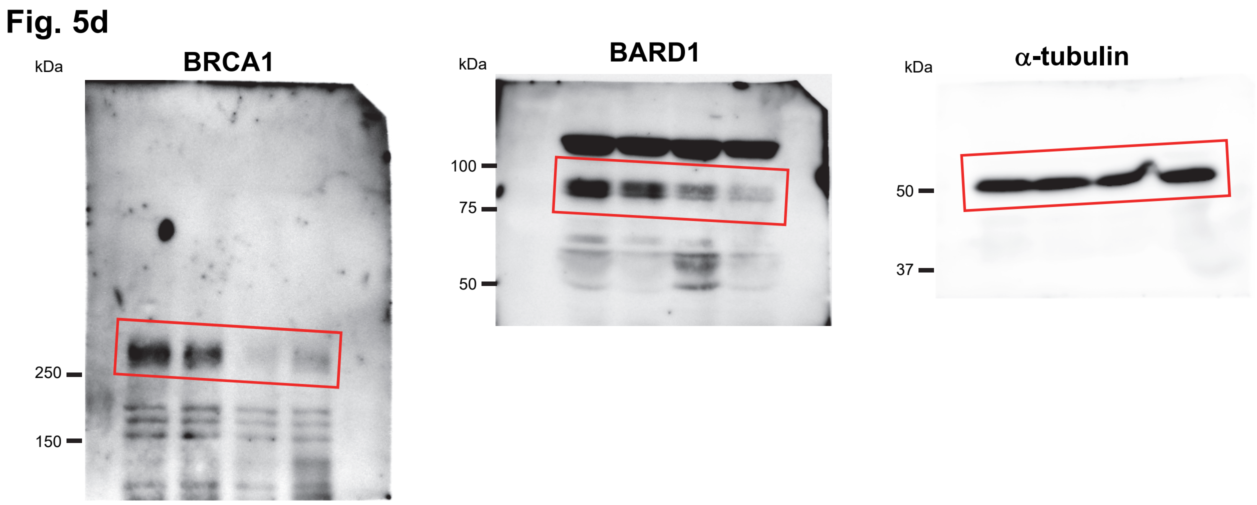

Supplementary Figure S6.
Uncropped images of Western blotting of Fig. 5d.

Supplementary Figure S7

Fig. 6a

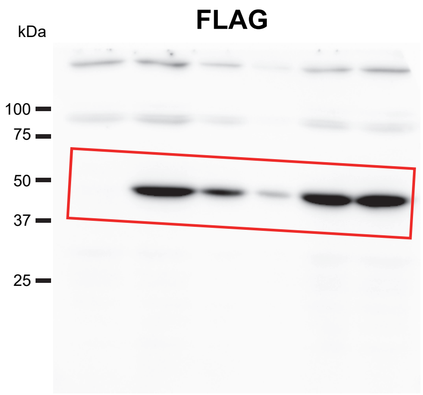

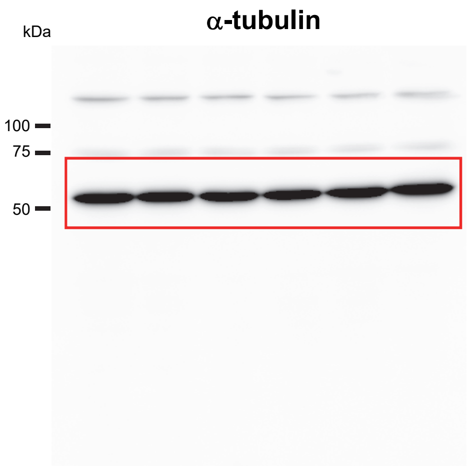

Supplementary Figure S7.
Uncropped images of Western blotting of Fig. 6a.

Supplementary Figure S8

Supplementary Fig. S1a

Fig. 3a

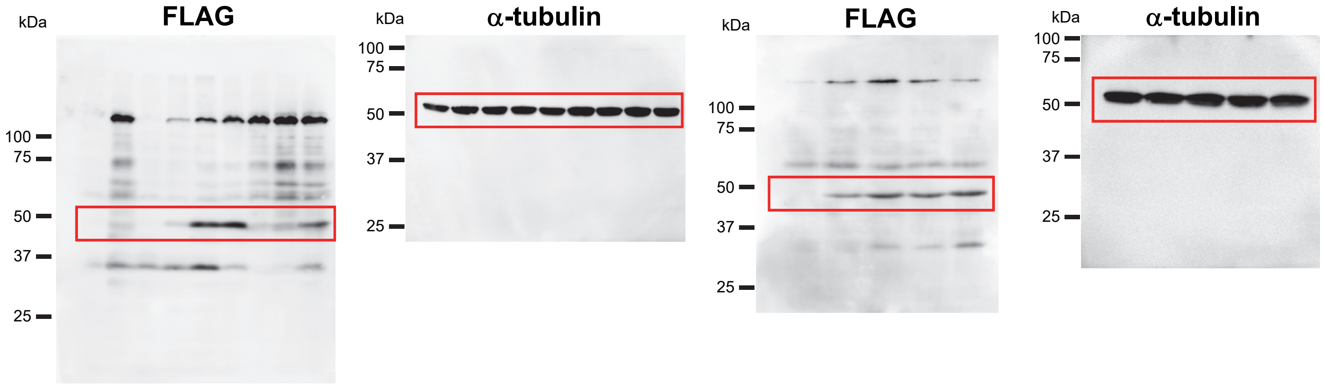

Supplementary Fig. S1b

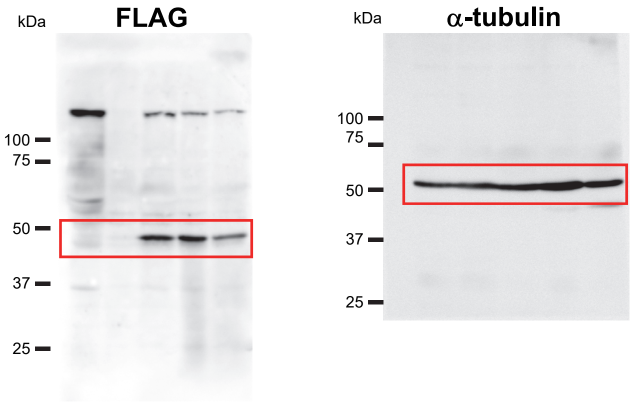

Supplementary Fig. S1c

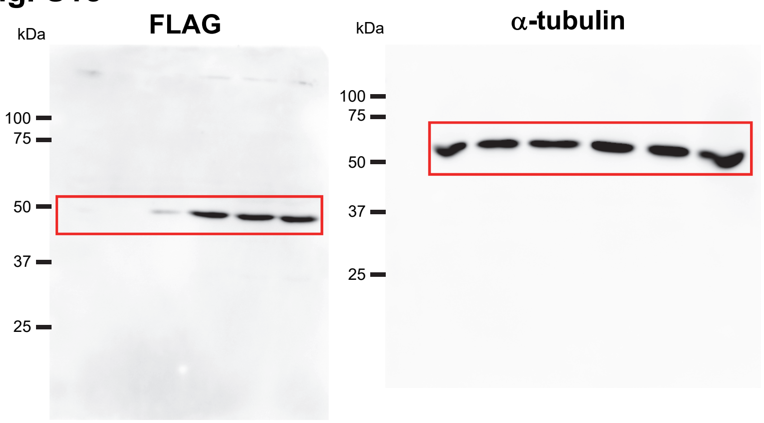

Supplementary Figure S8.
Uncropped images of Western blotting of Supplementary Fig. 1a, b, and c.

Supplementary Figure S9

Supplementary Fig. S2

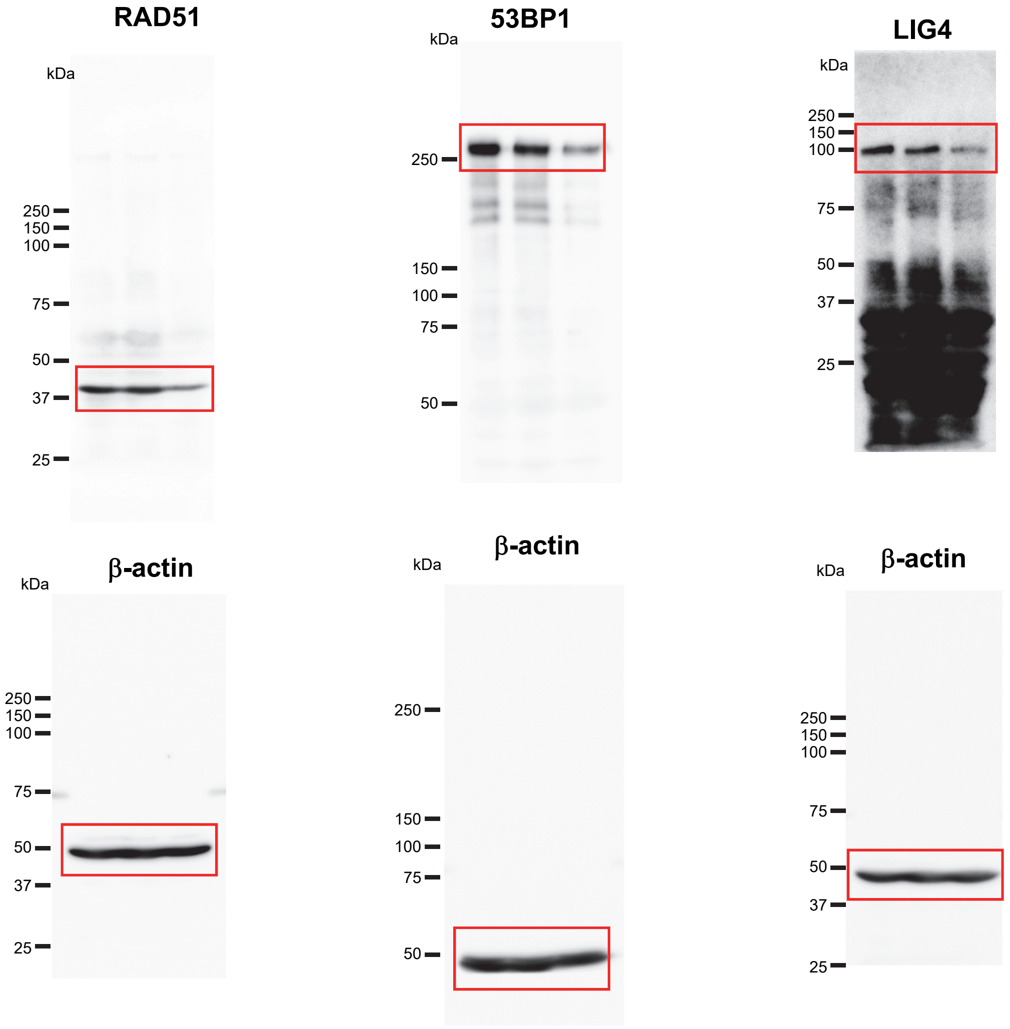

Supplementary Figure S9.
Uncropped images of Western blotting of Supplementary Fig. S2.
